# Seasonal Dynamics, Host Network Structure, and Environmental Drivers of Gamasid Mite Communities on Small Mammals in a Dry–Hot Valley: A Year-Round Monitoring Study

**DOI:** 10.3390/ani16192999

**Published:** 2026-09-23

**Authors:** Peng-Wu Yin, Yan Lv, Wen-Yu Song, Rong Fan, Cheng-Fu Zhao, Zhi-Wei Zhang, Ya-Fei Zhao, Wen-Ge Dong, Xian-Guo Guo

**Affiliations:** Yunnan Provincial Key Laboratory for Zoonosis Control and Prevention, Key Laboratory of Ectoparasite Systematics and Evolution of Yunnan Provincial Education Department, Institute of Pathogens and Vectors, Dali University, Dali 671000, China; pengwuyin@vip.163.com (P.-W.Y.);

**Keywords:** ectoparasite, community structure, small mammal host, seasonal dynamics, host mediation, environmental driving factor

## Abstract

Ectoparasitic gamasid mites on small mammals play crucial roles in transmitting zoonotic pathogens like hantaviruses associated with hemorrhagic fever with renal syndrome (HFRS). To elucidate their community, network structure, and seasonal drivers in xeric habitats, a consecutive 12-month localized survey was conducted in Waxi Village, Binchuan County, a dry–hot valley in Yunnan Province, southwest China, from November 2020 to October 2021. From 1329 captured small mammals (18 species, 89.62% rodents), 12,075 gamasid mites belonging to 33 species were identified. The community exhibited overwhelming dominance by the family Laelapidae (*C_r_* = 99.35%) and genus Laelaps (*C_r_* = 97.37%). *Laelaps algericus*, *L. guizhouensis*, and *L. nuttalli* constituted the dominant mite species (cumulative *C_r_* = 88.96%) and formed a tightly correlated core species cluster. Overall infestation prevalence (*P_M_*), mean intensity (*MI*), and mean abundance (*MA*) were 46.05%, 19.73 mites/host, and 9.09 mites/host, respectively. The bipartite host–gamasid network revealed a significantly nested architecture (NOD_Fobs_ = 31.16, *Z* = 14.03, *p* < 0.001) with high specialization (*H*_2_′ = 0.599), anchored by dominant hosts (*Rattus andamanensis*, *Apodemus chevrieri*, and *Mus caroli*). α-diversity analysis showed that Margalef richness, Shannon–Wiener index, and Gini–Simpson index peaked in autumn (November), while troughs occurred in mid-spring (April) and mid-autumn (October). Non-metric multidimensional scaling (NMDS, stress = 0.0048) and PERMANOVA (*R*^2^ = 0.074, *p* = 0.001) confirmed significant seasonal shifts in community composition. Standard redundancy analysis (RDA) suggested temperature as the primary climatic driver (*F* = 7.205, *p* = 0.001; total model explanation = 8.2%). However, partial RDA (pRDA) conditioning on host species identity demonstrated that climatic variables directly accounted for only 0.1% of net variance (*F* = 0.662, *p* = 0.521). Our findings indicate that gamasid mite community assembly in this dry–hot valley is characterized by strong species dominance and network nestedness and that seasonal fluctuations are consistent with a host-mediated pattern rather than being directly governed by climate.

## Simple Summary

Gamasid mites are small external parasites that live on rodents and can transmit serious human diseases, such as hemorrhagic fever with renal syndrome. To understand what drives their activity, we conducted a year-long field study in the dry–hot valley of Yunnan, southwest China. We found that mite populations are strongly concentrated on a few dominant rodent hosts, with their numbers peaking in autumn and reaching their lowest levels in winter and spring. Importantly, our findings show that local weather (such as temperature) affects mite numbers mainly by influencing their host animals, rather than affecting the mites directly. By identifying when and on which animals these mites thrive, this study provides useful information for surveillance and vector management to target rodent and vector management effectively, helping to reduce the risk of vector-borne disease outbreaks in local communities.

## 1. Introduction

Rodents and some other sympatric small mammals (e.g., shrews and tree shrews) are critical reservoir hosts for a variety of zoonotic diseases, and they commonly harbor gamasid mites and other ectoparasites on their body surfaces. Taxonomically, gamasid mites belong to the suborder Gamasina, order Parasitiformes within the subclass Acari of the phylum Arthropoda. Previous studies have demonstrated that several genera within Gamasina, such as *Laelaps*, *Haemolaelaps*, and *Ornithonyssus*, hold substantial medical and veterinary importance. Certain species within these genera can serve as transmission vectors for some zoonoses (zoonotic diseases), including rickettsial pox and hemorrhagic fever with renal syndrome (HFRS). These vectors are capable of transmitting pathogens not only among small mammals but also from these wildlife hosts to humans and domestic animals [1,2,3]. The transmission of zoonotic diseases like HFRS involves multiple intertwined components, such as natural foci, host animals, and vectors. The community structure, seasonal dynamics, and host selection preferences of ectoparasitic gamasid mites may substantially influence the efficiency and risk of pathogen transmission along the “host–vector” chain [2,4,5]. For ectoparasites like gamasid mites, specific geographical environments, host-related variables, climatic factors, and interspecific interactions can exert profound impacts on the parasite communities and their seasonal fluctuations [6,7]. In subtropical mountainous regions with pronounced seasonal variations, climatic factors such as temperature and humidity represent key ecological driving forces shaping the spatiotemporal patterns of gamasid mite communities [8].

Compared with mesic environments characterized by relatively abundant precipitation and mild climatic variables, xeric environments—such as dry–hot valleys marked by high evapotranspiration, prolonged seasonal drought, and elevated temperatures—impose stringent climatic constraints on ectoparasites [8]. While mesic environments typically sustain a relatively stable ectoparasite species composition with gradual seasonal transitions, xeric habitats are expected to foster sharper seasonal turnovers, heightened population aggregation, and stronger host specificity due to the heavy reliance of parasites on host habitats [9,10]. Consequently, gaining a deeper insight into how ectoparasitic gamasid mite communities respond to climatic regimes in xeric ecosystems is crucial for predicting pathogen transmission risks across distinct geographical landscapes.

To explore the community structure and seasonal dynamics of ectoparasitic gamasid mites within a specific geographical landscape, a 12-month consecutive localized field survey was conducted at a fixed survey site (Waxi Village, Binchuan County) situated in a dry–hot valley of Yunnan Province, southwest China, from November 2020 to October 2021. On the basis of elucidating the infestation status, species composition, basic community structure, and seasonal dynamics of gamasid mites on small mammals in this unique dry–hot valley environment, this study further aims to investigate the host–gamasid network relationships, the influence of host-related factors on the gamasid community structure, and the driving effects of climatic factors on the seasonal dynamics of the mite community. Yunnan Province, located in southwest China, is not only one of the crucial natural foci for zoonoses such as HFRS but also features highly complex and diverse topographical landforms and climatic regimes. Consequently, it has emerged as a prominent hotspot for research on small mammals and their associated ectoparasitic mites [9,10,11]. Conducting comprehensive ecological and community studies on gamasid mites associated with small mammals in Yunnan Province yields highly significant ecological and epidemiological implications.

Specifically, this study refines key aspects of ectoparasite community assembly in dry–hot valley ecosystems by testing three hypotheses: (1) seasonal dynamics in gamasid mite diversity are governed by a multi-factorial cascade, in which macro-environmental drivers (e.g., temperature and rainfall) primarily operate indirectly via host-mediated pathways—such as seasonal fluctuations in host density, behavior, and habitat structural complexity; (2) host–gamasid interaction networks in xeric habitats exhibit nestedness, specialized structure, largely shaped by dominant host species; and (3) climatic conditions across different seasons intensify the strong dominance of stress-tolerant gamasid species, resulting in structural shifts between species richness and community evenness. By elucidating infestation patterns, network architecture, and environmental drivers over a full annual cycle, this study offers novel ecological perspectives on parasite community assembly in xeric habitats and provides empirical evidence for monitoring vector-borne diseases in dry–hot valley foci.

## 2. Materials and Methods

### 2.1. Study Site and Field Survey

A 12-month consecutive field investigation was conducted at a fixed survey site, Waxi Village (25°43′ N, 100°24′ E), in Binchuan County, Dali Bai Autonomous Prefecture, Yunnan Province of southwest China, from November 2020 to October 2021 (Figure 1). The survey site is situated in a southern subtropical dry–hot valley, which belongs to a typical subtropical monsoon climate zone. The annual mean temperature is 18.3 °C, the annual relative humidity is 63%, and the annual average precipitation is 560.4 mm. The annual average evaporation reaches 2518.5 mm, which is approximately 4.4 times the annual precipitation. The high evaporation and low precipitation contribute to a unique climate characteristic of “high temperature, low humidity, and strong evaporation” in the valley region of Binchuan County [12,13]. To explore the seasonal dynamics of the gamasid mite community, the whole year was divided into four seasons: spring (March to May), summer (June to August), autumn (September to November), and winter (December to February of the following year) [7]. The monthly meteorological data (temperature, humidity, and precipitation) were obtained from the National Tibetan Plateau/Third Pole Environment Data Center (https://data.tpdc.ac.cn) [14].

### 2.2. Host Trapping and Mite Collection

Every month, 150–200 wire mouse cages (18 × 12 × 9 cm; Guixi Mousetrap Apparatus Factory, Guixi, Jiangxi, China) baited with food were placed at the fixed survey site to capture rodents and other sympatric small mammal hosts. The mouse cages were set in the late afternoon or evening and checked the following morning [13,15]. To ensure an adequate sample size of animal hosts, the duration of the field survey varied from 7 to 10 days per month, ensuring that no less than 100 hosts were captured each month. The captured small mammal hosts were deeply anesthetized, and all gamasid mites on their body surfaces were examined and collected over a large white tray according to a standardized procedure. The specimens were preserved in 70% or 75% ethanol [16]. Following ectoparasite collection, the animal hosts were identified on-site based on their external morphology (body size, shape, and coat color) and various measurements (body length, body weight, tail length, ear height, hind foot length, etc.), and subsequently humanely euthanized via cervical dislocation under deep anesthesia in strict accordance with approved veterinary protocols, with animal carcasses disposed of safely via biohazard incineration [16,17]. All animal experiments involved in this study were officially approved by the Animal Ethics Committee of Dali University (Approval No. DLDXLL2020-1104) and strictly followed the guidelines for animal welfare and protection.

### 2.3. Taxonomic Identification of Gamasid Mites

Gamasid mites fixed and preserved in 70% or 75% ethanol were rinsed 2–3 times with clean or distilled water to remove non-mite impurities. The rinsed gamasid mites were mounted with Hoyer’s solution to make slide specimens. After dehydration, transparency, and drying in an electric oven, each gamasid mite specimen was carefully examined, microscopically measured, and morphologically compared under an optical microscope. With the aid of taxonomic monographs, literature, and identification keys for gamasid mites, every specimen was identified to the species level [1,18,19,20]. Specimens that could not be accurately identified to the species level due to damage, debris coverage, blurred morphology, or being suspected new species were recorded as “unidentified species”, and these specimens were excluded from the statistical analysis of this study. The specimens of gamasid mites and representative small mammals are deposited in the Institute of Pathogens and Vectors, Dali University.

### 2.4. Indices of Infestation and Community Structure

The following infestation indices of gamasid mites were routinely calculated: (1) constituent ratio (*C_r_*): the percentage of a specific mite species out of the total number of all collected gamasid mites; (2) prevalence (*P_M_*): the percentage of infested hosts out of the total number of examined hosts; (3) mean abundance (*MA*): the average number of gamasid mites per examined small mammal host; (4) mean intensity (*MI*): the average number of gamasid mites per infested small mammal host [10,21,22].

The community structure indices were calculated using R software (v4.4.3) with its ecological extension package “vegan” (version 2.6-10) [23,24]:

(1) *α*-diversity: Margalef Richness, Shannon–Wiener diversity index, Gini–Simpson index and Pielou’s Evenness were calculated for each season. Seasonal differences in gamasid mite diversity indices (Margalef richness, Shannon–Wiener index, Gini–Simpson diversity, and Pielou’s evenness) were evaluated using non-parametric statistical methods. Pairwise comparisons between seasons (Spring, Summer, Autumn, and Winter) were performed using the Wilcoxon rank-sum test (with normal approximation for tie correction).
(2) *β*-diversity: Non-metric multidimensional scaling (NMDS) analysis was performed based on the Bray–Curtis distance matrix, and permutational multivariate analysis of variance (PERMANOVA, Adonis) was employed to test the driving effects of seasonal variation on community structure. The goodness of model fit was evaluated by the stress value (stress), and the differences in coordinate values along each NMDS axis across seasons were compared using the Kruskal–Wallis test. To test whether PERMANOVA results were influenced by differences in within-group multivariate dispersion across seasons, a multivariate homogeneity of groups dispersions test was performed using the betadisper() function in the vegan package, with statistical significance evaluated via ANOVA (*F*-test) and permutation tests (permutest).

### 2.5. Host–Gamasid Mite Bipartite Network

To reveal interaction patterns between animal hosts and gamasid mites, a bipartite ecological network matrix was constructed using host species (*n* = 12 host species; 6 uninfested or singleton rare host species without mite counts were excluded) as lower-level nodes and gamasid mite species (*n* = 33) as upper-level nodes, weighted by interaction frequency (parasitic abundance). Network analysis was implemented using the R package bipartite (v2.18) [25]. Global network topology was quantified using the networklevel() function, calculating Connectance (*C*), Weighted Nestedness (NODF based on overlap and decreasing fill), and Network Specialization (*H*_2_′). To evaluate whether the observed nestedness (NODF_obs_) significantly deviated from random expectation, a null model test was conducted using nullmodel (method = 4) with 1000 randomized network permutations, maintaining marginal totals (row and column sum constraints). Standardized effect sizes (*Z*-score) and empirical *p*-values were calculated (*Z* = (NODFobs-mean (NODF_null_))/SD (NODF_null_)). At the node level, degree, normalized degree, species strength, species specificity index (*d*’), and nested rank were computed via specieslevel(). A bipartite Sankey flow diagram was generated using the networkD3 package (version 0.4.1) to visualize host–parasite flow pathways [26].

### 2.6. Co-Occurrence and Environmental Drivers

(1) Correlation analysis: Gamasid mites with a total abundance threshold of >50 individuals were selected for interspecific association analysis (*n* = 12 dominant species). The exact sampling unit used for calculating the correlation matrix was the individual host sample (*n* = 1329). Abundance data per sampling unit were log(x + 1)-transformed to meet normality assumptions and stabilize variance. Pairwise Pearson correlation coefficients (*r*) were computed among the 12 species. To account for inflation of Type I errors due to evaluating 66 pairwise correlations, *p*-values were adjusted for multiple testing using the Benjamini–Hochberg (BH) False Discovery Rate (FDR) procedure. Hierarchical clustering heatmap analysis was performed using the “pheatmap” package (version 1.0.12) in R, with statistical significance annotated based on FDR-adjusted *p*-values (*p_adj_ ≤ 0.05, ** *p*_adj_ ≤ 0.01, *** *p*_adj_ ≤ 0.001) [27].
(2) Redundancy analysis (RDA) was employed to evaluate the explanatory power of climatic variables (mean monthly temperature, relative humidity, and precipitation) on the seasonal structural dynamics of the ectoparasitic gamasid mite community. Unlike the α-diversity analysis, which utilized only infested hosts (*n* = 612), the species abundance matrix for RDA was constructed using all trapped host animals as rows (*n* = 1329) to properly accommodate absolute zero-abundance states, followed by Hellinger transformation to reduce the influence of double zeros and extreme values. The environmental matrix consisted of the corresponding monthly climatic metrics repeatedly assigned to the host individuals captured in each respective month. To properly accommodate the nested sampling design and address potential pseudoreplication arising from shared monthly climatic records across individual hosts within the same month, statistical significance was evaluated using a block-permutation scheme (*n* = 999). Specifically, permutations were executed by exchanging entire monthly climatic vectors across sampling months (i.e., permuting monthly time blocks using how(plots = Plots(strata = Month)) in the vegan package) rather than permuting individual host observations within months. This permuted distribution provides a statistically valid inferential test for climatic predictors that vary exclusively between months. Permutations were constrained within sampling months as strata/blocks using the how(plots = Plots(strata = Month)) function in the vegan package in R. Marginal effects of individual environmental variables and the overall constrained ordination model were tested via permutation tests (*p* < 0.05). All ordination graphics were rendered using the ggplot2 package (version 4.0.3) [28].

### 2.7. Host-Mediated Effects

Partial Redundancy Analysis (pRDA) and Host Control. To disentangle the direct climatic driving effects on the gamasid mite community from host-mediated confounding variations, a partial redundancy analysis (pRDA) was conducted. Given that gamasid mites exhibit strong host specificity, seasonal fluctuations in host community composition could potentially confound the perceived environmental effects. Therefore, host species identity was incorporated into the pRDA model as a categorical covariate (Condition (Host_Species)) to condition out the variance explained by host identity. The net explanatory power and marginal statistical significance of climatic variables (temperature, humidity, and rainfall) in the pRDA model were evaluated using the same block-permutation scheme (*n* = 999) permuted across sampling months while conditioning on host species identity. The net explanatory power and marginal statistical significance of climatic variables (temperature, humidity, and rainfall) were subsequently evaluated using restricted permutation tests (*n* = 999) stratified by sampling month [29].

## 3. Results

### 3.1. Species Composition of Small Mammal Hosts and Ectoparasitic Gamasid Mites

During a fixed-site investigation conducted in Waxi Village (25°43′ N, 100°24′ E), Binchuan County, Yunnan Province, from November 2020 to October 2021, a total of 1329 small mammal hosts (rodents and some other sympatric small mammals) were captured. These hosts belonged to three orders (Rodentia, Eulipotyphla, and Scandentia), five families, 12 genera, and 18 species. Among them, the order Rodentia comprised 11 species (*C_r_* = 61.11%, 11/18) and exhibited an absolute dominance in individual abundance (*C_r_* = 89.62%, 1191/1329). Of the 18 small mammal species captured, *Rattus andamanensis*, *Apodemus chevrieri*, and *Mus caroli* were the most abundant, accounting for 27.54%, 26.71%, and 22.05% of the total captures, respectively. Together, these three dominant host species comprised 1014 individuals (*C_r_* = 76.30%, 1014/1329) (Table 1, Figure 2).

A total of 12,075 gamasid mites were collected and identified from the body surface of the 1329 hosts, representing 33 species, 11 genera, and three families. The highest numbers of gamasid mite species were collected from *R. andamanensis* (20 species) and *A. chevrieri* (18 species). Meanwhile, *A. chevrieri* and *M. caroli* harbored the largest numbers of mite individuals, with their combined mite abundance accounting for 78.38% (9464/12,075) of the total mite abundance (Table 1). A sunburst chart (Figure 3) was utilized to visualize the hierarchy and constituent ratios of the 12,075 gamasid mites across different taxonomic levels (family, genus, and species). At the family level, Laelapidae exhibited the highest proportion (*C_r_* = 99.35%, 11,996/12,075). At the genus level, the genus *Laelaps* was predominant (*C_r_* = 97.37%, 11,757/12,075). At the species level, three species emerged as the dominants: *Laelaps algericus* (*C_r_* = 68.42%; 8262/12,075), *Laelaps guizhouensis* (*C_r_* = 10.58%; 1278/12,075), and *Laelaps nuttalli* (*C_r_* = 9.95%; 1202/12,075). These three dominant species jointly constituted 88.96% of the total mite abundance (*C_r_* = 88.96%, 10,742/12,075) (Table 2, Figure 3). Among the 33 species collected, *Ornithonyssus bacoti*, *Haemolaelaps glasgowi*, and *Laelaps echidninus* are known vectors or potential vectors of hemorrhagic fever with renal syndrome (HFRS) (Table 2).

### 3.2. Infestation Status of Gamasid Mites

Pooling the data across all 12 months at the Waxi survey site, the overall infestation indices of gamasid mites on all small mammal hosts were: prevalence (*P_M_*) = 46.05%, mean abundance (*MA*) = 9.09 mites/host, and mean intensity (*MI*) = 19.73 mites/host. Among the 33 identified gamasid mite species, *L. algericus*, *L. guizhouensis*, and *L. nuttalli* were the most abundant dominant species (*C_r_* = 88.96%) (Table 3), all of which displayed relatively high infestation indices (*P_M_*: 26.86%, 2.71%, 11.66%; *MA*: 6.22, 0.96, 0.90; *MI*: = 23.14, 35.50, 7.75, respectively) (Table 3). Three of the 33 identified species serve as vectors or potential vectors of HFRS, namely *O. bacoti*, *L. echidninus*, and *H. glasgowi* (Table 2). Notably, *O. bacoti* and *L. echidninus* also maintained relatively high infestation intensity (*MI* = 16.50 for *O. bacoti*; *MI* = 9.38 for *L. echidninus*).

### 3.3. Community Diversity Analysis

A total of 1329 small mammal hosts were captured during the year-round monitoring period at the Waxi survey site. The majority of the 1329 hosts were free of gamasid mite infestation, with the overall infestation rate being 46.05%, and only 612 hosts harbored gamasid mites, including 159 in spring, 179 in summer, 150 in autumn, and 124 in winter. To ensure an accurate evaluation of the spatiotemporal structural characteristics of the gamasid mite community, only the 612 positive hosts were selected as the valid hosts for the community analysis, and the 717 negative hosts without gamasid mite infestation (1329 − 612 = 717) were excluded from the community dataset. There were 12,075 individual gamasid mites (belonging to 33 species) identified from the 612 valid hosts (Table 4).

To elucidate the monthly dynamic succession of core species within the gamasid mite community, a species abundance heatmap was constructed (Figure 4). The map displays the log-transformed monthly abundance of the 33 remaining species, with the vertical axis representing the species and the horizontal axis representing the sampling months (Months 1–12). The color intensity reflects the mite abundance transformed by log (Abundance + 1), transitioning from pale yellow (low abundance/not detected) to dark red (high abundance). The results indicated that most gamasid mite species reached higher abundances during the rainy season (June–September). Dominant species such as *L. algericus* peaked in summer, showing a trend toward seasonal outbreaks. Conversely, some species (e.g., *Androlaelaps singularis* and *Androlaelaps hsui*) exhibited lower abundances during the dry season.

*Laelaps algericus* was distributed year-round, and its abundance remained at exceptionally high levels (log-value > 6) from March to October, forming the ecological core of the local gamasid mite community. The diversity and total abundance of the community began to climb rapidly in spring (March), peaked at the transition between summer and autumn, and subsequently declined significantly from November to January of the following year due to falling temperatures.

Monthly analysis of *α*-diversity indices revealed distinct seasonal fluctuations in the Margalef richness, Gini–Simpson, Shannon–Wiener, and Pielou’s evenness indices of the gamasid mite community. The Margalef richness index exhibited a bimodal fluctuation pattern, with the primary peak occurring in late autumn (November: 2.41) and secondary peaks in summer (August: 1.86; June: 1.68), whereas the lowest values were recorded in mid-spring (April: 0.88) and early winter (December: 1.03) (Table 4). The Gini–Simpson and Shannon–Wiener indices shared highly consistent trajectories, generally characterized by higher values in autumn and winter and lower values in spring and summer. Both indices peaked in November (0.79 and 1.96, respectively) and remained relatively elevated in January and February, while reaching their lowest points in April (0.29 and 0.63, respectively), with additional pronounced troughs observed in June and October (Figure 5). In contrast, Pielou’s evenness index was asynchronous with the richness index, attaining maximum values in November (0.74), February (0.68), and January (0.65), and minimum values in June and October (0.27) (Table 5). Notably, despite species richness reaching its maximum of 14 species in June, the evenness index remained remarkably low, indicating the strong dominance of particular superabundant species (e.g., *Laelaps algericus*) within the summer community.

The *α*-diversity of the ectoparasitic gamasid mite community on small mammal hosts exhibited significant seasonal variations (Figure 6). Based on the distribution characteristics in the violin plots and box plots, as well as the results of the Wilcoxon rank-sum test, the specific seasonal dynamics of each diversity index were as follows: Gamasid mite species richness (Margalef richness) peaked in autumn, being significantly higher than in spring, summer, and winter (*p* < 0.01; Figure 6A), whereas no statistically significant differences in species richness were observed among spring, summer, and winter. Similarly, the Shannon–Wiener diversity index reached its highest level in autumn, being significantly higher than in spring (*p* < 0.001) and winter (*p* < 0.01; Figure 6B); the index in summer ranked second but remained significantly higher than in winter (*p* < 0.01). The Gini–Simpson diversity index demonstrated a marked prominence in autumn, with values remarkably higher than those in spring (*p* < 0.0001), summer (*p* < 0.001), and winter (*p* < 0.01; Figure 6C), indicating that the gamasid community in autumn achieved the highest overall diversity and a relatively even dominance distribution. Pielou’s evenness index remained at relatively high levels during autumn and winter, with community evenness in autumn being significantly higher than in spring (*p* < 0.01; Figure 6D), while no significant differences were detected among the other seasons. Synthesizing the various *α*-diversity indices, the ectoparasitic gamasid mite community on small mammals in this region exhibited a seasonal succession pattern characterized by “peaking in autumn, remaining suppressed in winter and spring, and gradually recovering in summer.” As the core period for community flourishing, autumn supported significantly higher species richness, overall diversity, and community evenness compared to other seasons.

In this study, non-metric multidimensional scaling (NMDS) analysis based on Bray–Curtis distance was performed on the gamasid mite community. In Figure 7, colored scatters represent individual gamasid mite community samples collected from small mammal hosts across different seasons. The diamond-shaped color blocks denote the centroids of the communities for each season. Black vector rays represent the top eight dominant gamasid mite species that contributed most to the variance in community structure, in which the length and direction of each ray reflect the influence of that species’ abundance on the ordination of the samples. Beta diversity analysis indicated that seasonal succession is a pivotal driver shaping the structure of the gamasid mite community on small mammal hosts. The NMDS ordination based on Bray–Curtis distance (Figure 7) yielded an exceptionally low stress value (stress = 0.0048), demonstrating the high reliability of the ordination results. Multivariate dispersion analysis revealed significant seasonal differences in within-group dispersion (*F* = 16.258, *p* < 0.001). This indicates that the observed seasonal variation in PERMANOVA (*R*^2^ = 0.074, *F* = 16.253, *p* = 0.001) reflects a combination of directional shifts in community centroids and differences in multivariate dispersion among seasons, with summer and autumn communities displaying tighter multivariate clustering compared to winter and spring.

Further Kruskal–Wallis tests on the NMDS axes revealed that seasonal differences were primarily reflected along the NMDS1 axis (*p* < 0.001), while the NMDS2 axis also demonstrated a statistically significant difference (*p* < 0.01). At the species level, the abundance vector of *Haemolaelaps orientalis* pointed toward the winter sample zone in the upper right of the ordination plot, indicating that this species dominated the winter community. In contrast, *Eulaelaps substabularis* pointed toward the fourth quadrant, demonstrating a strong correlation with summer and autumn communities. Species such as *O. bacoti*, *L. fukienensis*, and *H. liae* clustered tightly around the center of the ordination plot, reflecting their relatively balanced distribution across different seasons and forming the baseline framework of the gamasid mite community.

The Venn diagram showed that seven gamasid mite species were distributed across all four seasons, constituting the ecological core of the local gamasid mite community on small mammals (Figure 8). Pronounced asymmetry was observed in mite abundance and endemic species distribution among seasons. The summer community exhibited the highest total abundance (total abundance = 4731 mites) and contained five season-specific endemic species (*Haemolaelaps monticola*, *Cosmolaelaps miles*, *Androlaelaps pavlovskii*, *E. substabularis*, and *C. xiajiangensis*), with the dominant assemblage heavily led by *L. guizhouensis* (558 mites). The autumn community had a total abundance of 3160 mites, ranking second to summer, but its number of endemic species reached the annual peak with six species (including *H. glasgowi*, *H. chinensis*, and *H. cordatus*), where *L. algericus* (2335 mites) and *L. nuttalli* (460 mites) served as the dominant species. No endemic gamasid mite species were found in the spring community (total abundance of 2985 mites), which was composed entirely of species shared with other seasons and dominated primarily by *L. algericus* (1958 mites). In winter, the total abundance of the community dropped to the annual minimum (1199 mites), with only two endemic species preserved (*L. hongaiensis* and *H. orientalis*), and the dominance shifted toward the more cold-tolerant *L. nuttalli* (401 mites) (Figure 8).

### 3.4. Bipartite Relationships Between Gamasid Mites and Small Mammal Hosts

Among the 18 captured small mammal species, 12 host species with positive gamasid mite parasitism were incorporated into the bipartite host–gamasid network. The network exhibited a moderately loose overall linkage, with a global Connectance (*C*) of 0.237 and a high degree of complementary specialization (*H*_2_′ = 0.599), indicating that host–parasite interactions were highly structured rather than opportunistic. Statistical testing against 1000 Patefield null models confirmed that the network possessed a statistically significant nested structure (NODF_obs_ = 31.16, NODF_null_ = 10.82 ± 1.45, *Z* = 14.03, *p* < 0.001). This statistical validation demonstrates that specialist gamasid mites interact with a subset of the hosts occupied by generalist mites, rather than forming isolated compartments.

Figure 9 visually depicts the parasitic flow pathways between 12 small mammal host species and 33 gamasid mite species. The 12 host species exhibited distinct carrying capacities. The dominant host *Apodemus chevrieri* displayed the strongest output effect (parasitic load: 5069 mites), linking to 18 mite species. A highly specific dominant link was observed between *Mus caroli* and *Laelaps algericus* (4139 mites), constituting a major energy flow axis.

At the host level (Table 6), *Rattus andamanensis* occupied the central core position (degree = 20, normalized degree = 0.606, species strength = 9.413, nested rank = 0.000). At the mite level (Table 7), *Laelaps guizhouensis* and *Laelaps xingyiensis* acted as key generalist cores (nested rank = 0.000), whereas species like *Haemolaelaps orientalis* occupied absolute peripheral ranks (nested rank = 1.000).

### 3.5. Interspecific Relationships Among Gamasid Mites

Figure 10 presents a heatmap of interspecific relationships among major gamasid mites based on a Pearson correlation coefficient matrix, displaying the co-occurrence correlations among 12 major gamasid mite species with a total abundance exceeding 50 individuals. The color scale transitions from dark blue (strong negative correlation) to dark red (strong positive correlation). The dendrograms on the left and top classify the species based on the similarity of their correlations, revealing the modular structure within the gamasid mite community. The right annotation bar (White–Dark Green) represents the log-transformed abundance of each species. Asterisks within grid cells denote significance levels based on FDR-adjusted *p*-values (*p*_adj_ ≤ 0.001).

Pearson correlation matrix analysis demonstrated significant co-occurrence patterns within the ectoparasitic gamasid mite community on small mammals (Figure 10). Cluster analysis partitioned the 12 major gamasid mite species into two main associated clusters: (1) Primary positively associated cluster: centered around *L. algericus*, *L. guizhouensis*, *Laelaps paucisetosa*, and *L. xingyiensis*. Species within this cluster displayed strong positive correlations, particularly between *L. guizhouensis* and *L. paucisetosa* (*r* = 0.959, *p*_adj_ < 0.001), as well as between *L. algericus* and *L. guizhouensis* (*r* = 0.862, *p*_adj_ < 0.001) and *L. xingyiensis* (*r* = 0.824, *p*_adj_ < 0.001), all exhibiting robust positive correlation coefficients (*r* > 0.800). (2) Secondary positively associated cluster: comprising *Laelaps taingueni*, *L. nuttalli*, and *A. hsui*. Among them, *L. taingueni* and *A. hsui* exhibited exceptional synchrony (*r* = 0.958, *p*_adj_ < 0.001), while *L. nuttalli* and *A. hsui* showed a moderately strong positive correlation (*r* = 0.743, *p*_adj_ < 0.01).

Furthermore, *Ornithonyssus bacoti* served as an intermediate species connecting other gamasid mites, exhibiting positive associations with *L. taingueni* (*r* = 0.813, *p*_adj_ < 0.001) and *L. xingyiensis* (*r* = 0.684, *p*_adj_ < 0.01), thereby linking the two main clusters within the community matrix structure. In contrast, *Laelaps chini* displayed weak, non-significant negative correlations with most species (*r* < −0.1, *p*_adj_ > 0.5), while *H. liae* exhibited a high degree of independence within the co-occurrence network (*r* < −0.1, *p*_adj_ > 0.5).

### 3.6. Driving Effects of Environmental Factors on Gamasid Mite Community Structure

Figure 11 presents the redundancy analysis (RDA) ordination diagram of the ectoparasitic gamasid mite community structure on small mammal hosts in relation to climatic factors, illustrating the apparent effects of environmental variables (temperature, humidity, and rainfall) on community composition and their contributions to spatiotemporal succession. Individual host samples are represented by scatters, and the semi-transparent ellipses denote the 95% confidence intervals of the gamasid mite communities across different seasons. Thick orange arrows represent environmental factor vectors, whose lengths reflect their explanatory power on community variation, and their directions reflect their correlations with the ordination axes. Thin gray arrows represent vectors of the dominant gamasid mite species.

The global RDA model was statistically significant (*F* = 12.118, *p* < 0.001), with all constrained axes together explaining 8.2% of the total variation in the gamasid mite community. The first ordination axis (RDA1) and the second axis (RDA2) explained 7.4% and 0.7% of the variance, respectively. Forward selection and marginal effects tests demonstrated that among the three climatic factors, temperature exhibited the primary driving effect (*F* = 7.205, *p* = 0.001). In contrast, the marginal driving effects of humidity (*F* = 2.028, *p* = 0.129) and rainfall (*F* = 1.050, *p* = 0.309) were not statistically significant.

Regarding species–environment correlations, the distribution direction of Laelaps algericus was highly consistent with the temperature and rainfall vectors, indicating that its abundance increased markedly during warm, humid seasons or in high-temperature microhabitats. In contrast, the vectors for *L. nuttalli* and *H. musculi* ran opposite to the temperature gradient, suggesting potential tolerance for low-temperature conditions or a seasonal parasitic preference during colder months. The seasonal sample points displayed a distinct trajectory: summer and autumn samples extended along the positive direction of the RDA1 axis, correlating strongly with the temperature vector, whereas winter and spring samples clustered along the negative direction of RDA1, demonstrating high community similarity.

However, because unconditioned RDA cannot separate the intrinsic thermal sensitivity of ectoparasites from host population fluctuations, these apparent climatic effects require further validation to confirm whether temperature acts directly on gamasid mites or indirectly via host-mediated processes.

### 3.7. Host-Mediated Effects via Partial Redundancy Analysis (pRDA)

Seasonal trapping records indicated that the dominant host species (e.g., *Rattus andamanensis*, *Apodemus chevrieri*, and *Mus caroli*) maintained presence across all seasons, though their relative abundance varied temporally and spatially (Table 8/Figure 12). To disentangle true direct climatic impacts from indirect host-mediated mechanisms, partial RDA (pRDA) was performed by controlling for host species identity as a conditioning matrix. Crucially, after partialling out the baseline variance attributable to host identity, the net climatic model completely lost its statistical significance (*F* = 0.662, *p* = 0.521), accounting for a mere 0.1% of the net variance in the gamasid mite community. None of the individual climatic variables retained significant net marginal effects (temperature: *p* = 0.495; humidity: *p* = 0.545; rainfall: *p* = 0.569). These contrasting findings between unconditioned RDA and pRDA suggest that the strong “temperature driving effect” observed in standard RDA is largely an artifact resulting from host seasonal dynamics. Rather than climatic factors exerting direct environmental filtering on gamasid mites, the spatiotemporal assembly of these ectoparasitic communities is consistent with a host-mediated process, reflecting strong links between mite communities and host dynamics.

## 4. Discussion

The survey site of this study (Waxi Village) is situated in the transition zone between the central Yunnan Plateau and the Hengduan Mountains in southwest China. This area is characterized by an annual mean temperature of 18.3 °C, a perennial relative humidity of 63%, and an annual average rainfall of 560.4 mm, falling into the landscape category of a southern subtropical dry–hot valley [30,31]. To explore the community structure and seasonal dynamics of parasitic gamasid mites in this unique geographical environment, a 12-month consecutive localized monitoring survey was conducted in this dry–hot valley for the first time. The results showed that the overall infestation prevalence, mean intensity, and mean abundance of gamasid mites throughout the year were *P_M_* = 46.05%, *MI* = 19.73 mites/host, and *MA* = 9.09 mites/host, respectively. Among the 18 small mammal host species captured, three rodent species belonging to the order Rodentia, namely *Rattus andamanensis*, *Apodemus chevrieri*, and *Mus caroli*, collectively accounted for a dominant constituent ratio (*C_r_* = 76.30%). Among the 33 gamasid mite species identified, three species belonging to the genus *Laelaps* of the family Laelapidae, namely *Laelaps algericus*, *Laelaps guizhouensis*, and *Laelaps nuttalli*, had a combined *C_r_* = 88.96%. Results of the seasonal fluctuations in α-diversity indices of the gamasid mite community indicated that the community structure was most stable during winter and late autumn (November, January–February), characterized by an even distribution of individuals and the highest overall diversity. Influenced by factors such as host dynamics, both species richness and diversity reached their annual troughs in spring (April). In summer (June–August), although the absolute number of detected species increased numerically, this recovery in richness was not statistically significant compared to spring; furthermore, due to the prominent dominance of dominant species, community evenness and overall diversity remained relatively low.

Rigorous topological evaluation confirmed that the host–gamasid mite bipartite network exhibited a statistically significant nested structure (NODF = 31.16, *Z* = 14.03, *p* < 0.001) alongside high specialization (*H*_2_′ = 0.599). Rather than relying solely on visual inspection of the Sankey diagram (Figure 9), null model permutation tests statistically validated that specialized gamasid mites interact with a nested subset of hosts associated with core generalist mites. This core–periphery network topology indicates that a few generalist rodent hosts (e.g., *Rattus andamanensis*, *Apodemus chevrieri*) and dominant generalist mites (e.g., *Laelaps guizhouensis*, *Laelaps xingyiensis*) form the resilient structural backbone of the parasitic community. Previous studies have demonstrated that small mammal and arthropod communities in the dry–hot valleys of southwest China are mainly dominated by a few dominant species, representing a “few-species-dominated” community structure type, and the gamasid mite community on small mammals in this study is highly consistent with this pattern [32,33]. Among the 33 identified gamasid mite species, *Ornithonyssus bacoti*, *Laelaps echidninus*, and *Haemolaelaps glasgowi* are known vectors or potential vectors of hemorrhagic fever with renal syndrome (HFRS) [9,34,35]. Furthermore, the infestation indices for *O. bacoti* and *L. echidninus* were relatively high (*O. bacoti*: *MI* = 16.50; *L*. *echidninus*: *MI* = 9.38). Although these three vector mite species were not the dominant species in the community, their high infestation indices suggest a strong infesting capability across different host animals, which may possess certain epidemiological significance for the maintenance of zoonotic natural foci [2,36].

The non-metric multidimensional scaling (NMDS) ordination and PERMANOVA based on β-diversity revealed significant seasonal shifts in the gamasid mite community (*R*_2_ = 0.074, *p* = 0.001). Furthermore, the significant multivariate dispersion test (betadisper, *F* = 16.258, *p* < 0.001) complements the PERMANOVA findings. It demonstrates that seasonal dynamics involve not only subtle shifts in mean community composition (centroids) but also structural expansion and contraction of community variability (dispersion). Summer and autumn assemblages exhibited reduced dispersion, driven by the intense aggregation of superabundant species like *Laelaps algericus*, whereas winter and spring communities displayed wider multivariate spread as overall infestation rates declined. Consistent with these structural shifts, analysis of α-diversity indicated that species richness peaked in autumn, while troughs occurred in winter and spring. Specifically, the Shannon–Wiener diversity index exhibited a distinct temporal fluctuation with peaks in late autumn (November) and mid-winter (January), whereas pronounced troughs occurred in mid-spring (April) and October (Figure 5 and Figure 6). This temporary elevation in winter diversity is mainly attributed to the stable presence of cold-tolerant species (e.g., *L. nuttalli* and *H. orientalis*) paired with a drop in the absolute dominance of *L. algericus*, which likely accounts for the marked decline in mite diversity during other seasons [7,8]. Despite these pronounced seasonal fluctuations, the Venn diagram (Figure 8) demonstrated that seven core gamasid mite species persisted across all four seasons. These year-round resident species exhibit broad climatic tolerance and stable host utilization, thereby buffering the community against extreme seasonal turnover and maintaining its overall temporal stability throughout the year.

Correlation analysis demonstrated that the dominant mite species, centered around *L. algericus* and *L. guizhouensis*, formed a core positively associated species cluster within the community, with species within the cluster exhibiting a high positive correlation (*r* > 0.8) (Figure 10). The strong positive co-occurrence pattern among these core mite species suggests a high convergence in host preference or shared habitat utilization, indicating that they tend to co-infest the same host individuals or host species [37,38]. The formation of this core positively associated cluster reinforces the dominant role of a few major mite species in structuring the overall co-occurrence matrix of the ectoparasitic community.

The redundancy analysis (RDA) in this study indicated that the three climatic factors (temperature, humidity, and rainfall) collectively explained 8.2% of the total variation (*F* = 7.205, *p* = 0.001), with temperature contributing substantially to explaining the seasonal variation in the gamasid mite community. The integration of partial RDA provided pivotal insight into the assembly rules of ectoparasitic gamasid mite communities. While unconditioned ordination suggested seasonal shifts aligned with climate, removing host identity variance revealed that climatic factors alone account for negligible net variation (0.1%, *F* = 0.662, *p* = 0.521). This result suggests that the observed seasonal dynamics of gamasid mites are consistent with a host-mediated pathway—reflecting seasonal turnover in host abundance and availability—rather than indicating a direct climatic filtering effect on the mites [7,39,40]. While climatic stability in rodent burrows may buffer mites against external thermal stress, host physiological status and behavioral activity remain the primary direct filters. This strongly supports our refined hypothesis (Hypothesis 1), indicating that climatic factors do not directly filter the gamasid mite community, but rather shape seasonal vector diversity through a host-mediated cascade. Future studies incorporating host physiological parameters and multi-year tracking will further refine these host–parasite–environment interactomes.

*L. algericus*, *L. guizhouensis*, and *L. nuttalli* were the three dominant species at the survey site. However, due to various limitations such as manpower and financial resources, detection of relevant pathogens within these dominant mite species was not conducted in this study. Consequently, it is currently impossible to determine the epidemiological significance of these dominant mites in the transmission of zoonoses and the maintenance of natural foci, which constitutes a limitation of this study. In future research, it is necessary to incorporate pathogen detection for these core mite species to scientifically evaluate their epidemiological significance.

## 5. Conclusions

The ectoparasitic gamasid mite community on small mammals in the dry–hot valley of Waxi Village, Binchuan County, Yunnan Province, China, is co-dominated by a small number of core hosts and gamasid mites, which together form the structural skeleton of the host–gamasid interaction network. Within the order Rodentia, *Rattus andamanensis*, *Apodemus chevrieri*, and *Mus caroli* serve as the dominant host species, while *Laelaps algericus*, *Laelaps guizhouensis*, and *Laelaps nuttalli* within the genus *Laelaps* (family Laelapidae) constitute the dominant mite species. These three dominant gamasid mites form a core synergistic cluster, exhibiting highly convergent host preferences. The seasonal α-diversity of the gamasid mite community exhibited significant fluctuations, with its absolute peak occurring in late autumn (November) and secondary high values in mid-winter. Conversely, pronounced diversity troughs were observed during mid-spring (April) and mid-autumn (October); specifically, the Shannon–Wiener diversity index displays a distinct bimodal pattern in November and January. Regarding environmental drivers, although unconditioned redundancy analysis (RDA) initially suggested temperature as the primary climatic factor associated with seasonal shifts (explaining 8.2% of total variation), partial RDA (pRDA) conditioning on host species identity revealed that climatic variables directly accounted for a negligible net variation (0.1%, *p* = 0.521). This demonstrates that the observed seasonal fluctuations in gamasid mite communities in this dry–hot valley suggest a predominantly host-mediated process—reflecting seasonal turnover in host abundance and host availability—rather than being directly dictated by climatic conditions.

## Figures and Tables

**Figure 1:**
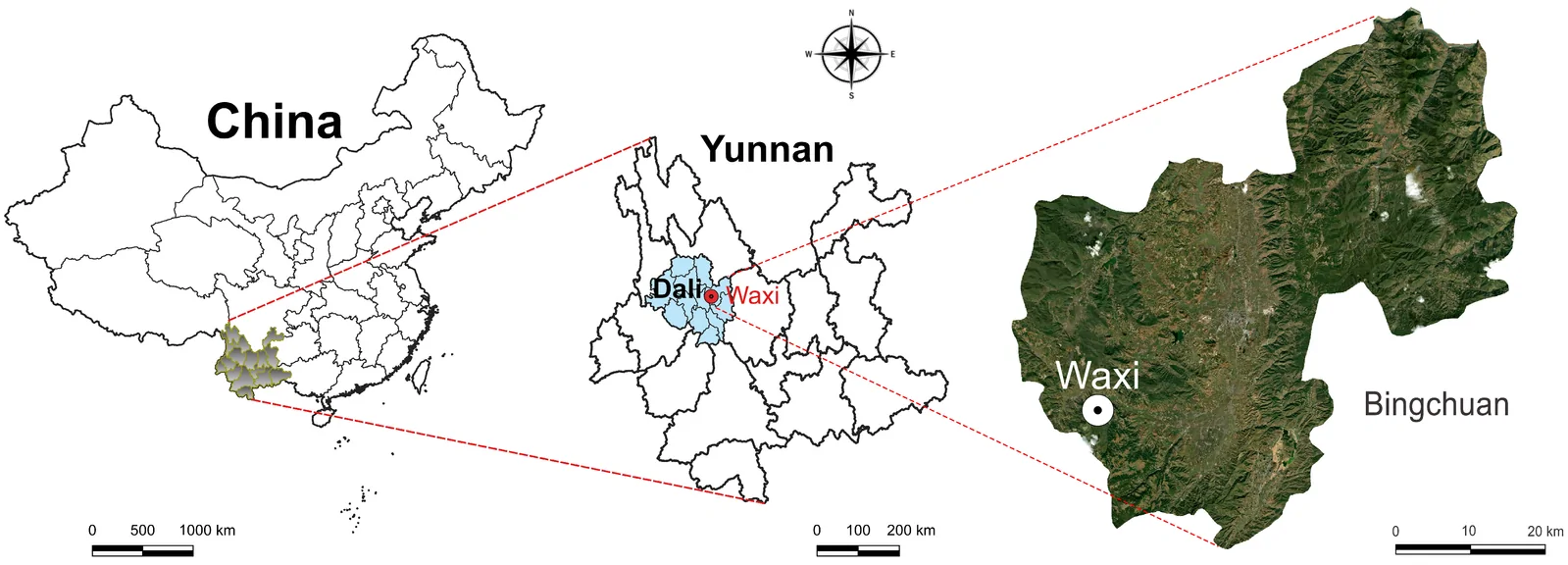
Map of China and Yunnan Province showing the location of the fixed survey site (Waxi village) in Binchuan County, Yunnan Province of southwest China (2020–2021).

**Figure 2:**
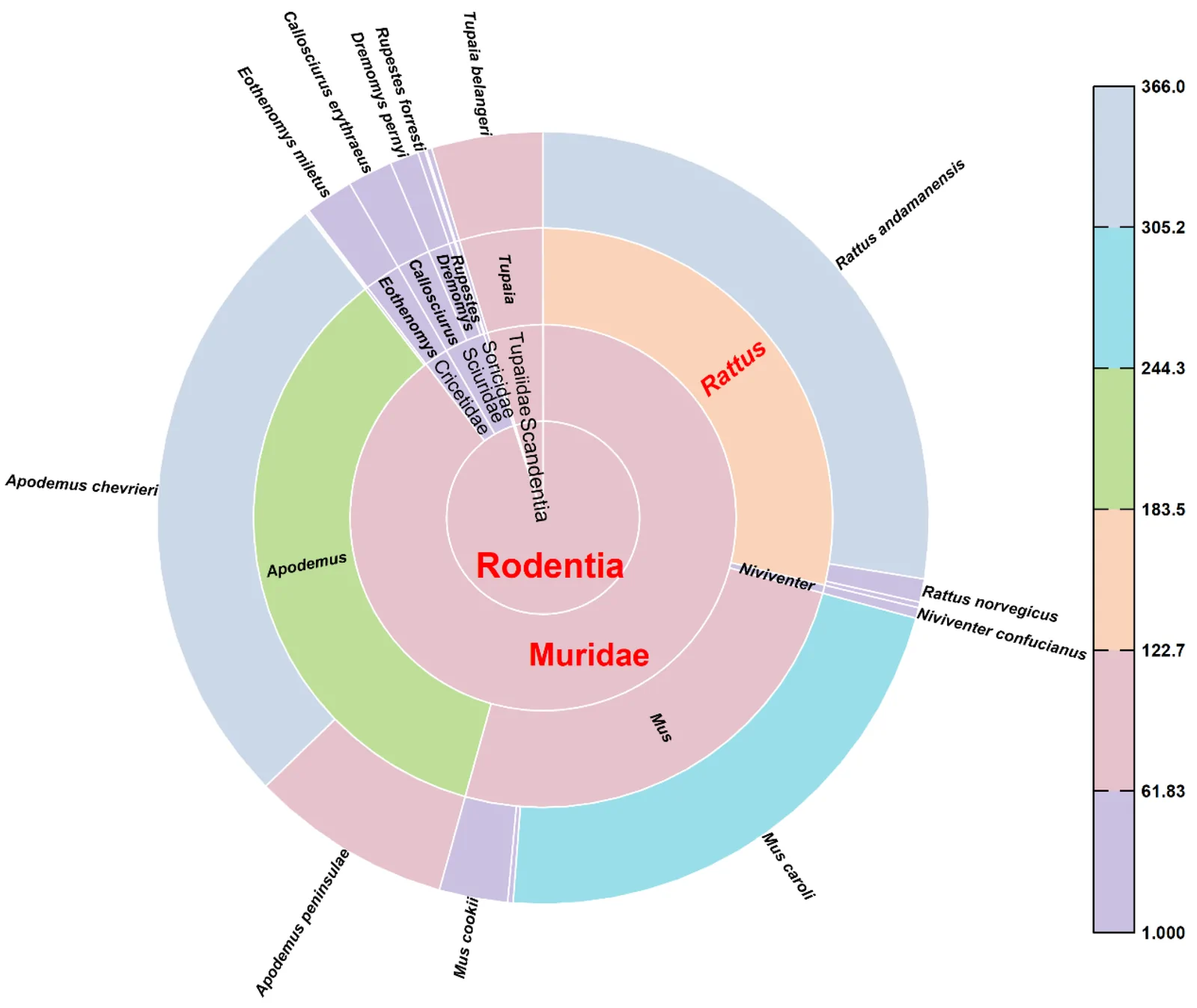
Sunburst chart illustrating the taxonomic hierarchy of host animals at the order, family, genus, and species levels. Annotation: Species labels representing less than 0.3% of the total count are omitted for clarity. These include *Micromys erythrotis* (1 individual) and *Micromys minutus* (1 individual) (Order Rodentia, Family Muridae, Genus *Micromys*); *Crocidura attenuata* (3 individuals) (Order Eulipotyphla, Family Soricidae, Genus *Crocidura*); and *Suncus murinus* (1 individual) (Order Eulipotyphla, Family Soricidae, Genus *Suncus*).

**Figure 3:**
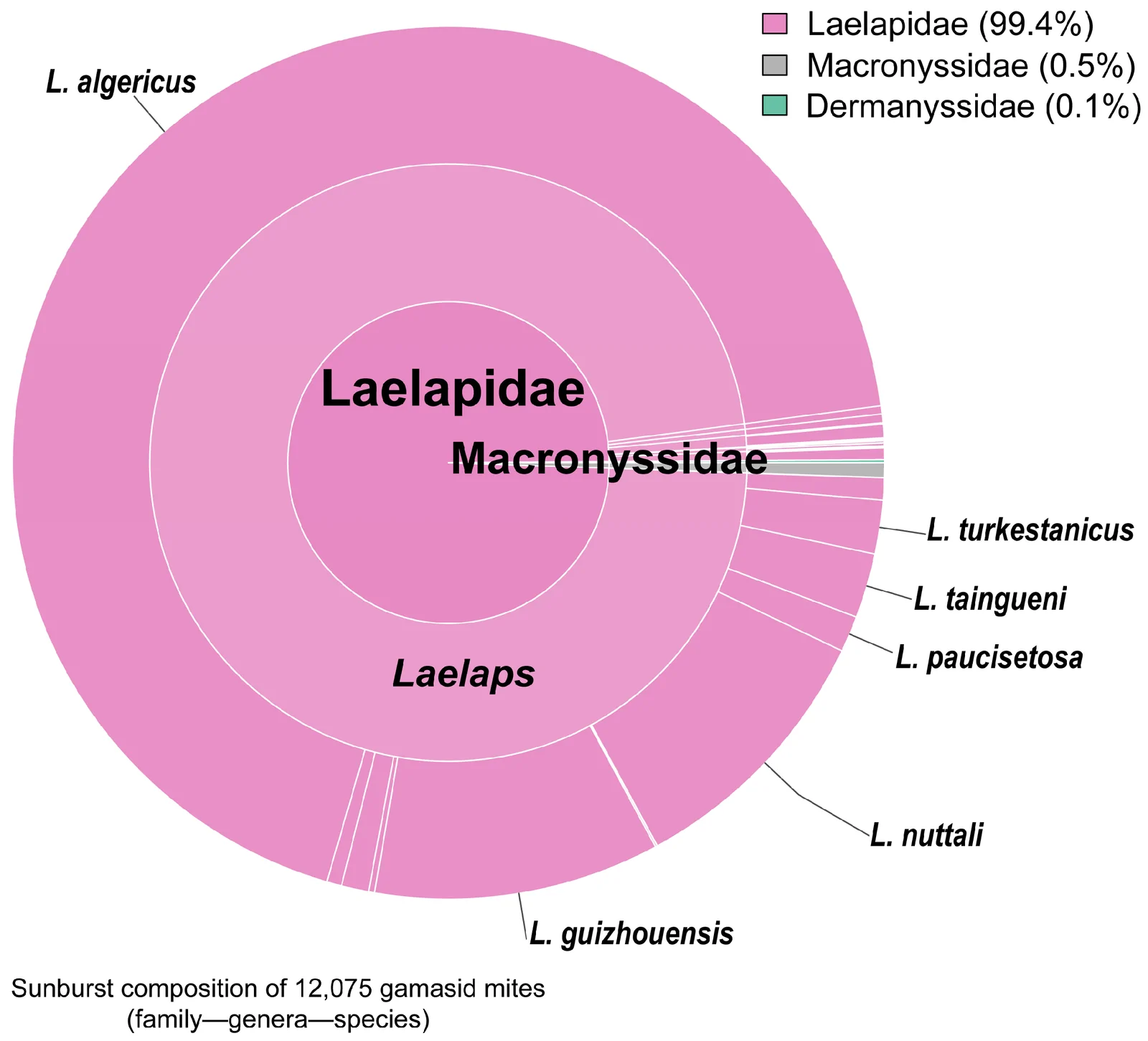
A sunburst map for visualizing the constituent ratios (*C_r_*) of gamasid mites (12,075 individuals) at different taxonomic levels (family, genera and species) at Waxi Village of Binchuan County, Yunnan Province of southwest China (2020–2021).

**Figure 4:**
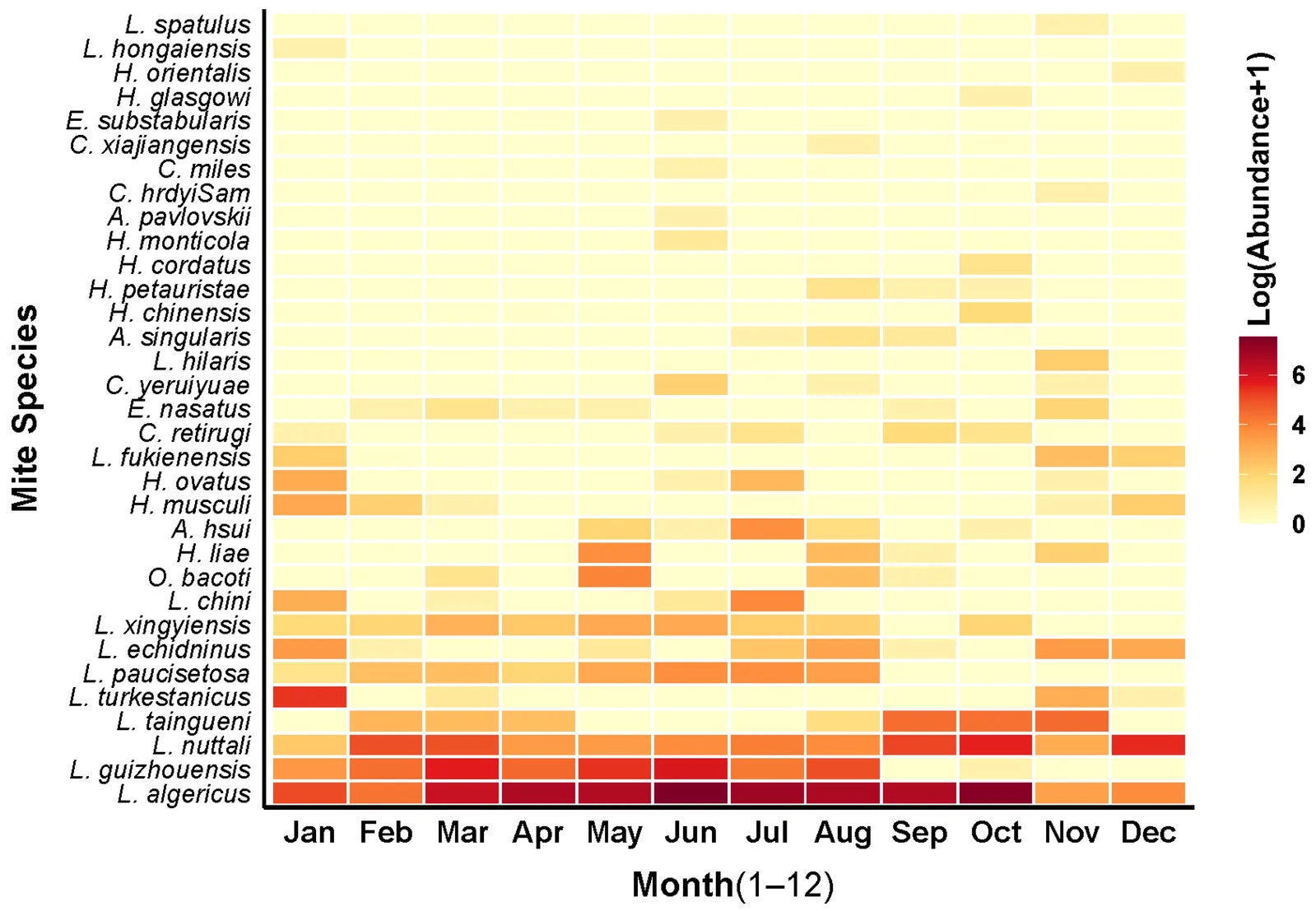
Monthly abundance patterns of common gamasid mite species at Waxi Village, Binchuan County, Yunnan Province, Southwest China (2020–2021). Annotation: Abundance data, derived from one year of continuous monitoring, reflect the absolute dominance of core species (such as *Laelaps algericus*) within the community and their population expansion during the warm season (March–October).

**Figure 5:**
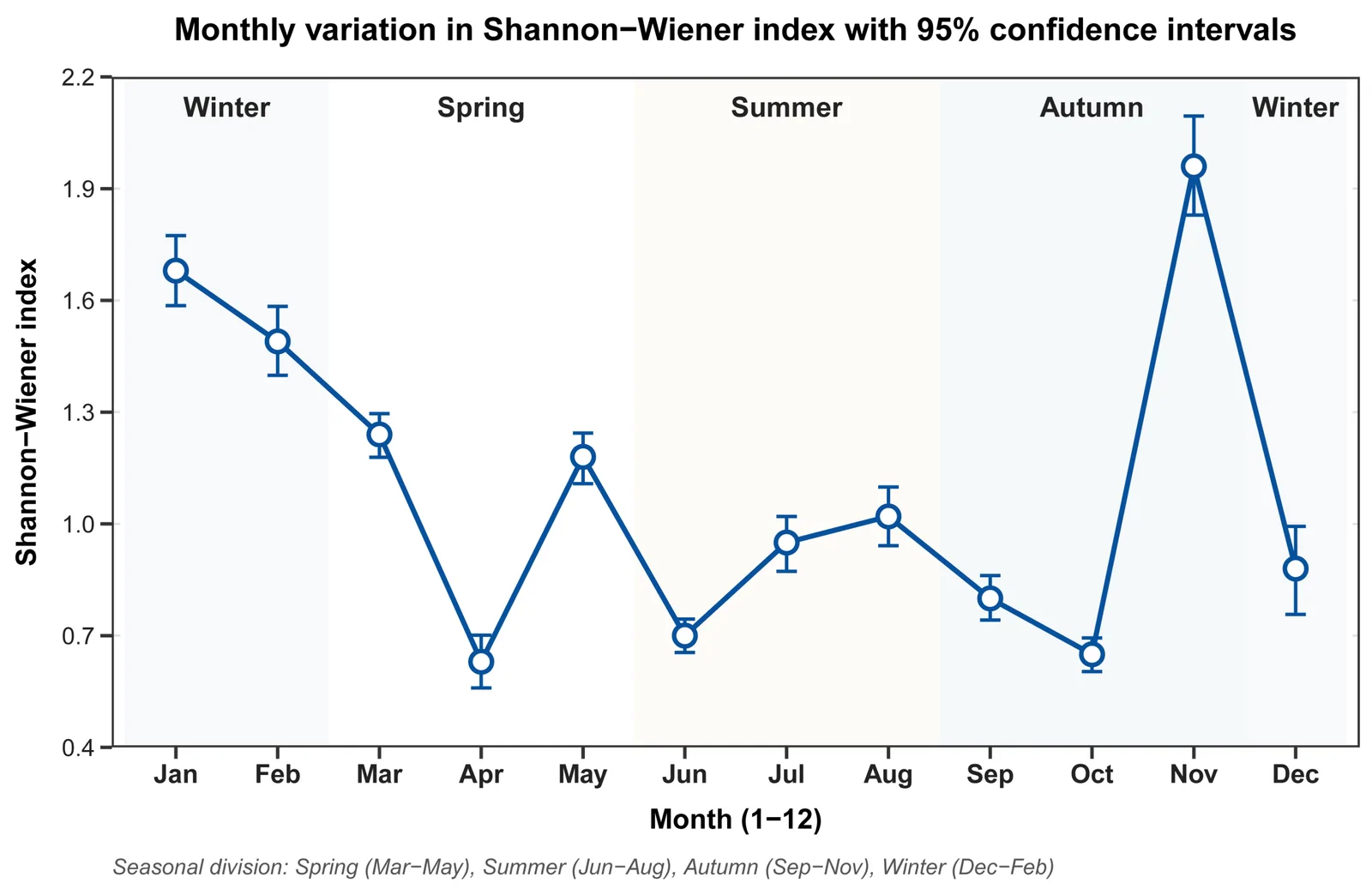
Monthly fluctuations of Shannon–Wiener index of gamasid mite community on small mammals at Waxi Village of Binchuan County, Yunnan Province of southwest China (2020–2021). Annotation: Shaded areas represent the 95% confidence interval. Background colors indicate seasonal divisions: Spring (March–May), Summer (June–August), Autumn (September–November), and Winter (December–February).

**Figure 6:**
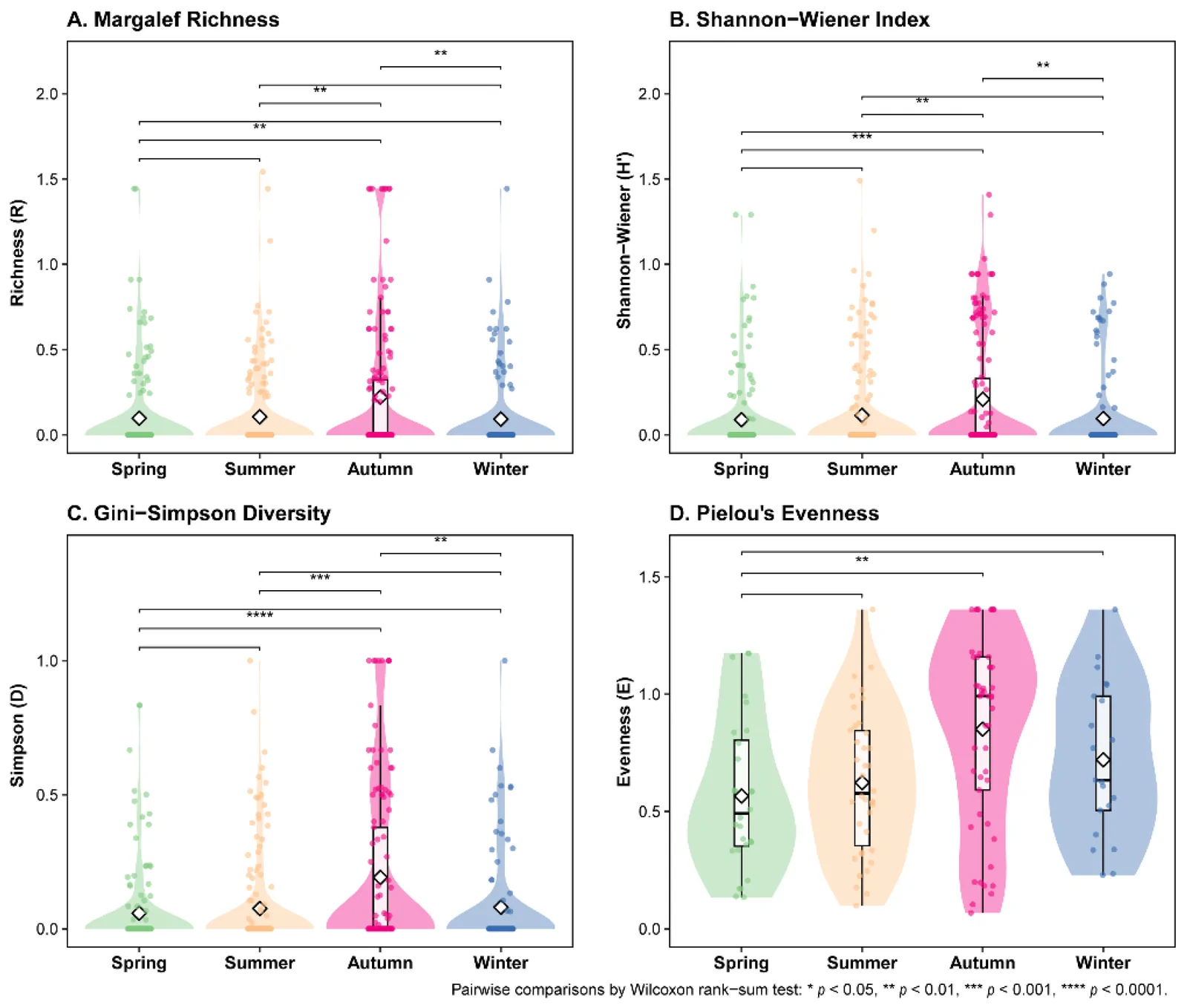
Seasonal variations in α-diversity indices of the gamasid mite community on small mammals at Waxi Village of Binchuan County, Yunnan Province of southwest China (2020–2021). Annotation: Statistical significance was defined at *p* < 0.05, with significance levels denoted as: * *p* < 0.05, ** *p* < 0.01, *** *p* < 0.001, and **** *p* < 0.0001. Non-significant differences (*p* ≥ 0.05) were omitted for clarity.

**Figure 7:**
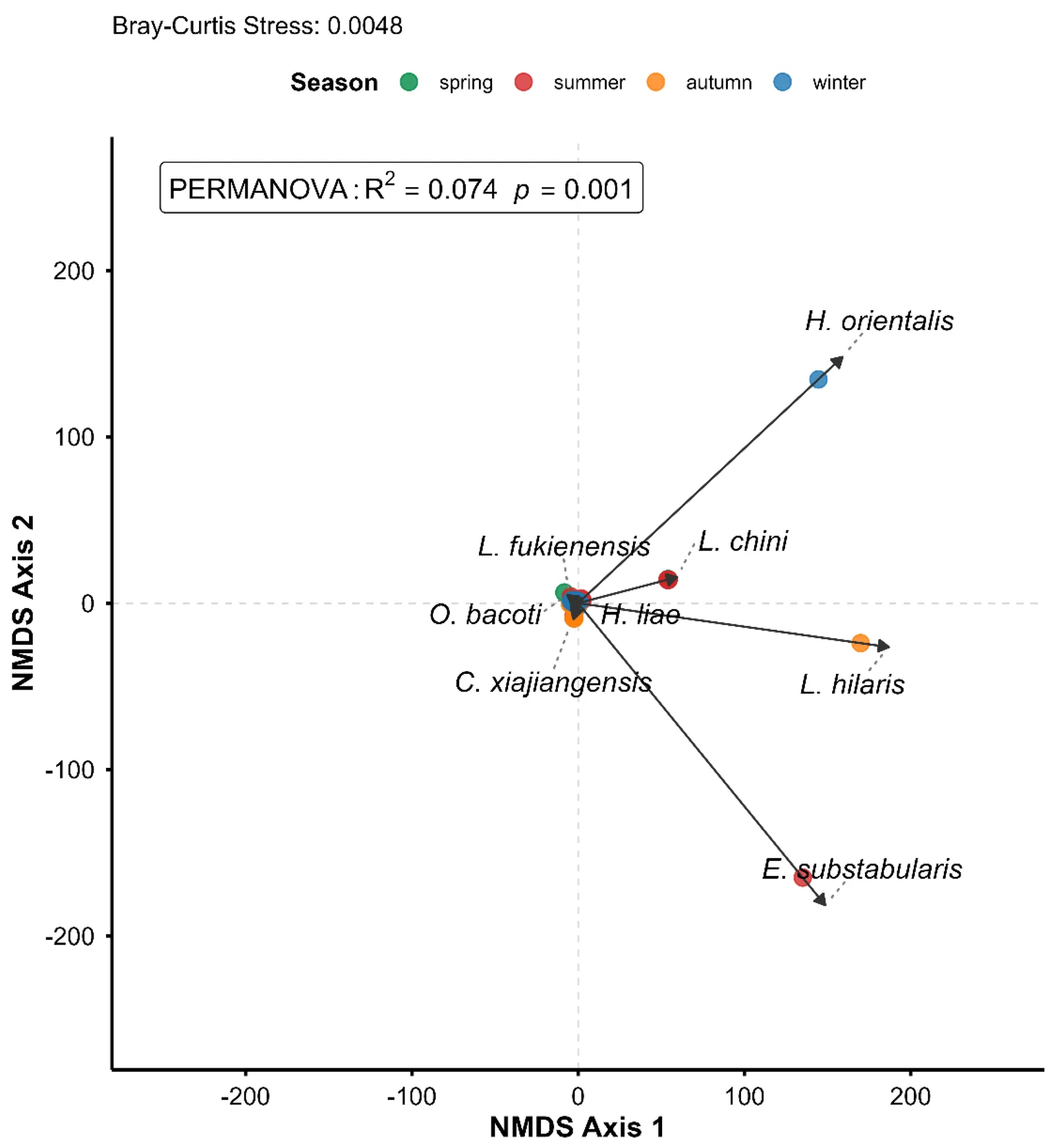
Non-metric multidimensional scaling (NMDS) of gamasid mite communities based on Bray–Curtis distance in different seasons at Waxi Village of Binchuan County, Yunnan Province of southwest China (2020–2021). Annotation: PERMANOVA test showed that seasonal factors had a significant effect on the community structure of gamasid mites (*R*^2^ = 0.074, *p* = 0.001); the Kruskal–Wallis test further confirmed that there were significant differences in both the first and second axes of NMDS among different seasons (*p* < 0.01).

**Figure 8:**
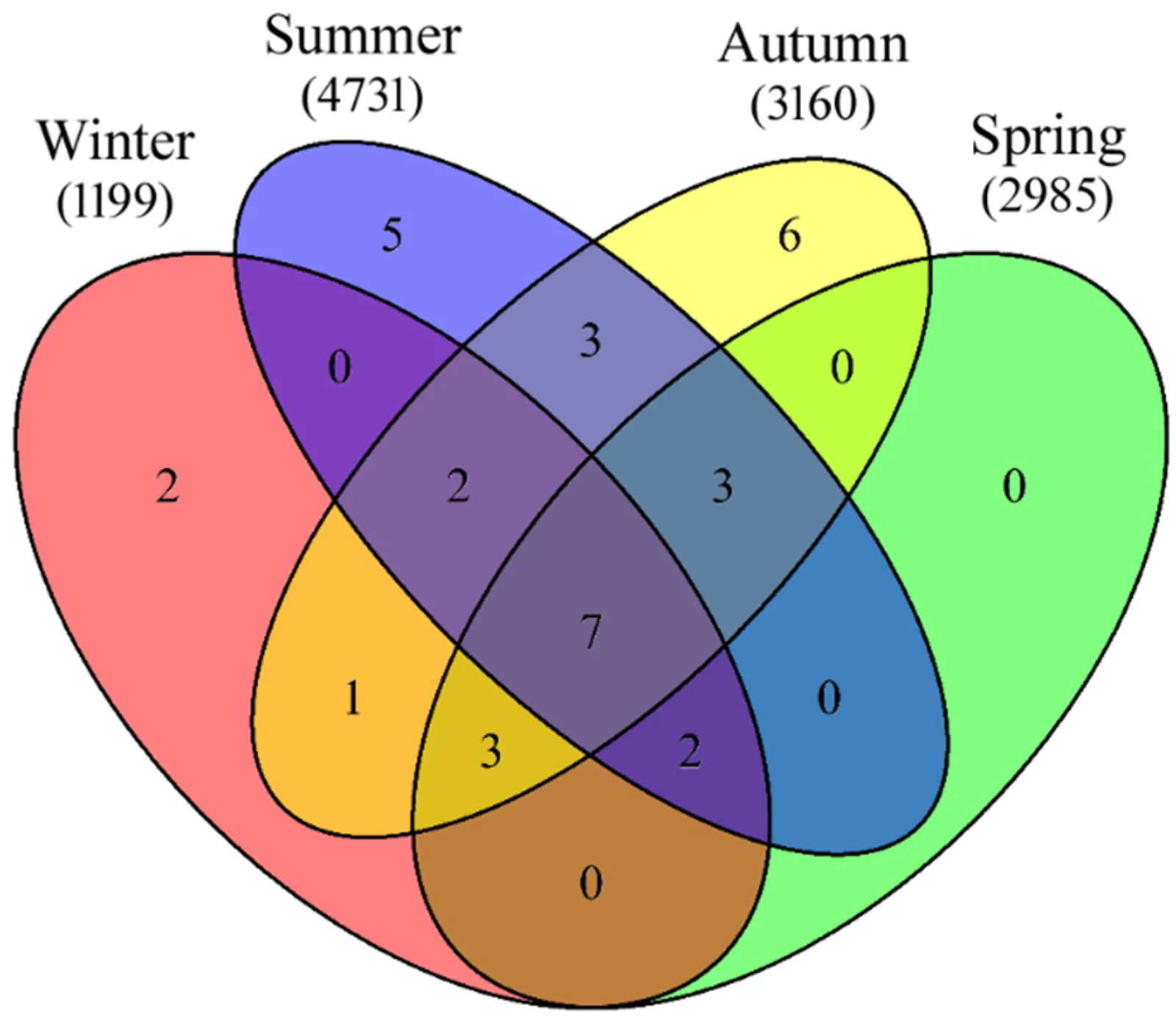
Venn diagram illustrating the seasonal distribution patterns of gamasid mite species community on small mammals at Waxi Village, Binchuan County, Yunnan Province, southwestern China (2020–2021). Annotation: The four circles represent spring, summer, autumn, and winter, respectively. Numerical values indicate the number of mite species recorded in each season. Shared species among two or more seasons are shown in the overlapping zones, while species unique to a single season are listed within the non-overlapping sectors of each circle. Total abundance per season is provided in parentheses beside each season label. The diagram reveals both seasonal overlap in dominant species and the presence of season-specific mite taxa.

**Figure 9:**
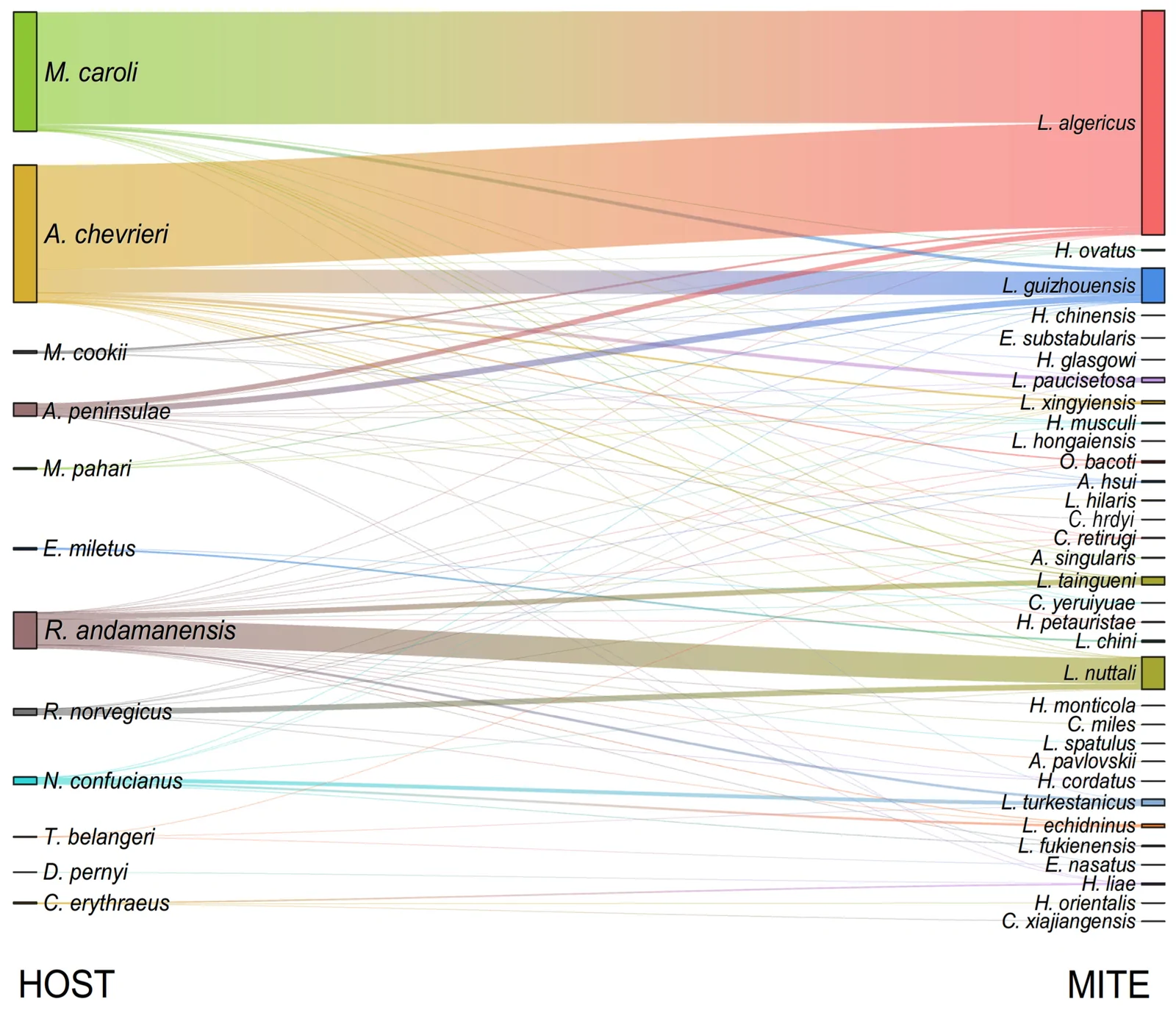
Bipartite relationships between 33 species of gamasid mites and 12 species of small mammal hosts at Waxi Village of Binchuan County, Yunnan Province of southwest China (2020–2021).

**Figure 10:**
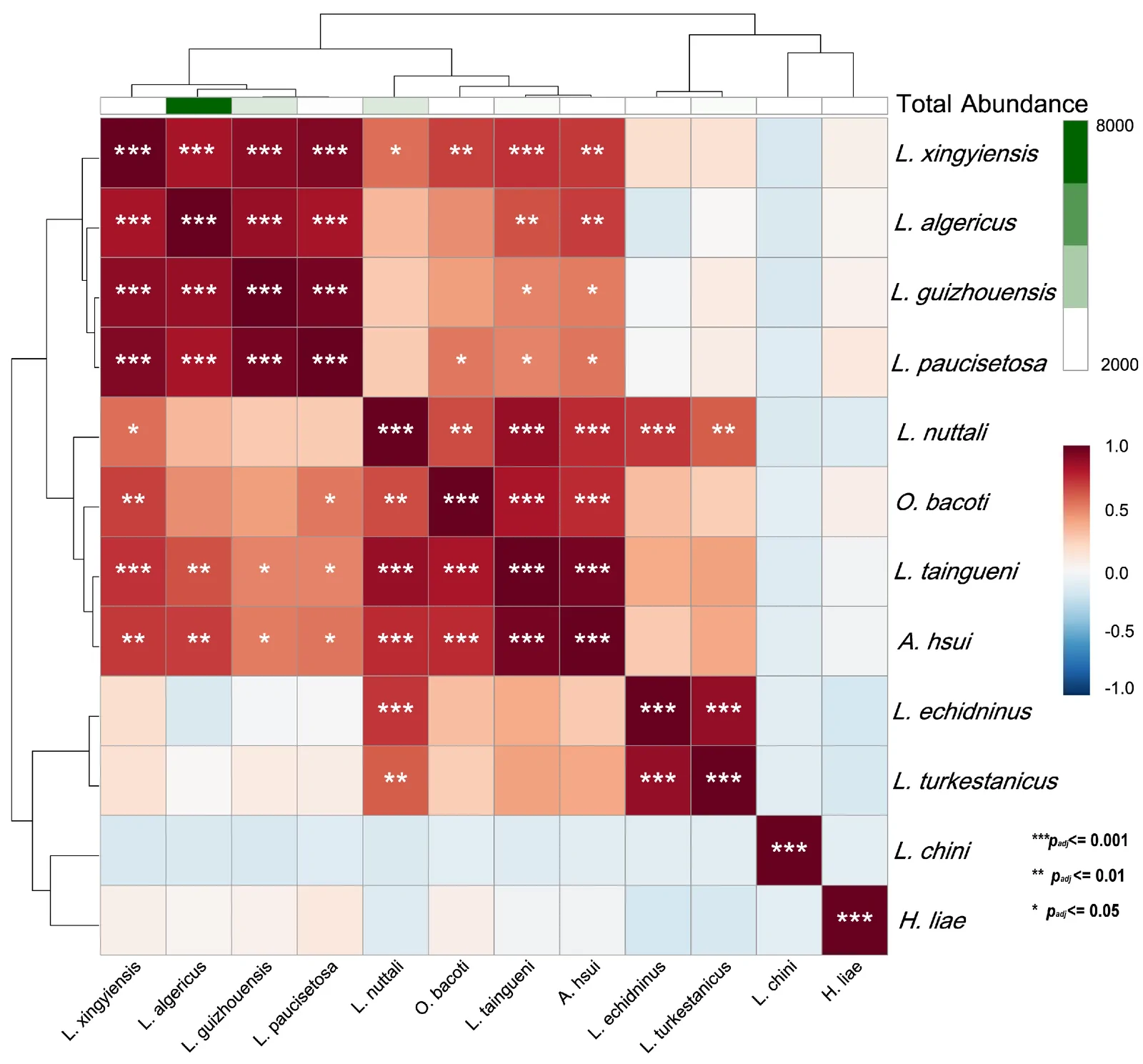
Heat map visualization for the relationship between any two of 12 main gamasid mite species at Waxi Village of Binchuan County, Yunnan Province of southwest China (2020–2021).

**Figure 11:**
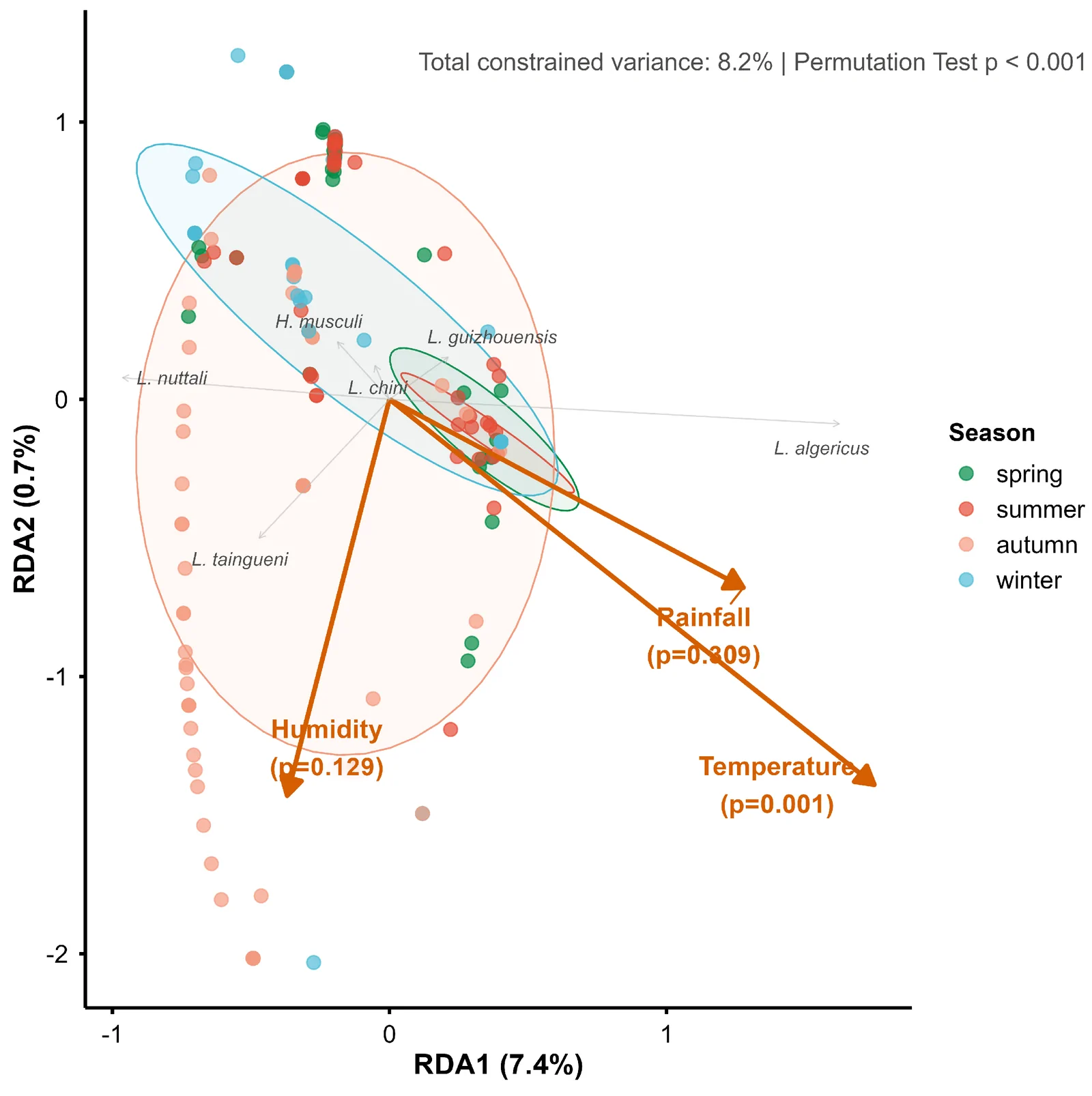
Redundancy analysis (RDA): environmental drivers on gamasid mite communities at Waxi Village, Binchuan County, Yunnan Province, Southwest China (2020–2021). Annotation: Redundancy analysis (RDA) ordination biplot showing the relationship between gamasid mite community composition and climatic variables. The global model is statistically significant (*F* = 12.118, *p* < 0.001, total constrained variance = 8.2%).

**Figure 12:**
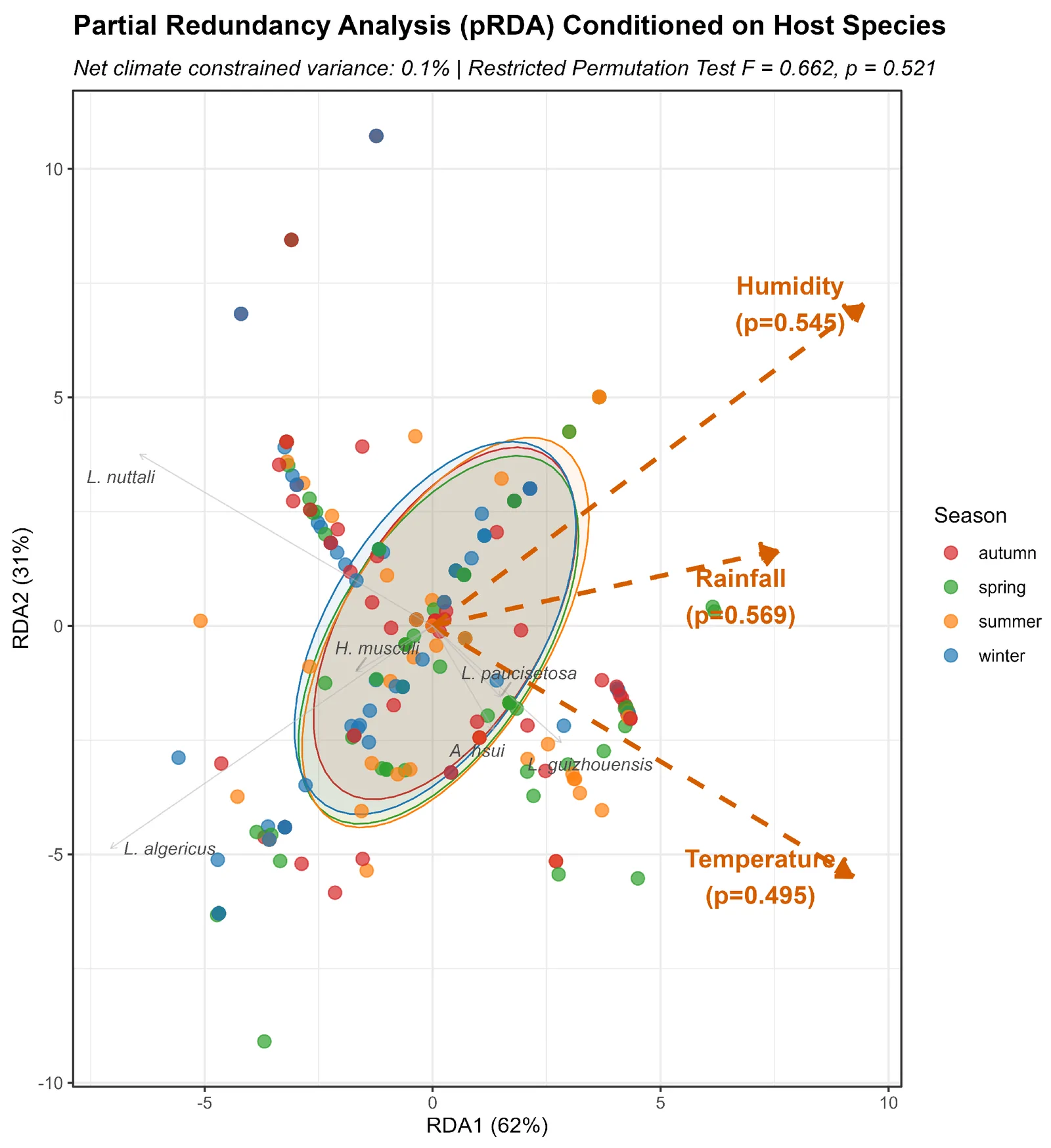
Partial redundancy analysis (pRDA) on gamasid mite communities at Waxi Village, Binchuan County, Yunnan Province, Southwest China (2020–2021).

**Table 1:** Dominant small mammal hosts and their ectoparasitic gamasid mites at Waxi Village of Binchuan County, Yunnan Province of southwest China (2020–2021).

Host Species	Individuals of Hosts		Species and Individuals of Gamasid Mites		
	NO.	*C_r_* (%)	Species	NO.	*C_r_* (%)
*Rattus andamanensis*	366	27.54	20	1342	11.11
*Apodemus chevrieri*	355	26.71	18	5069	41.98
*Mus caroli*	293	22.05	12	4395	36.40

**Table 2:** Taxonomic identification of gamasid mites on small mammal hosts at Waxi Village of Binchuan County, Yunnan Province of southwest China (2020–2021).

Taxonomic Taxa of Gamasid Mites	Collected Species and Individuals of Mites (The Figures Enclosed in Parentheses Are the Collected Individuals for Each Mite Species)
**Laelapidae**	11,996 individuals, 31 species, 9 genera
*Laelaps*	*L. algericus* (8262), *L. guizhouensis* (1278), *L. nuttalli* (1202), *L. taingueni* (289), *L. turkestanicus* (240), *L. paucisetosa* (162), *L. echidninus* * (122), *L. xingyiensis* (99), *L. chini* (67), *L. fukienensis* (27), *L. hilaris* (8), *L. hongaiensis* (1)
*Haemolaelaps*	*H. liae* (61), *H. petauristae* (5), *H. chinensis* (5), *H. cordatus* (3), *H. orientalis* (1), *H. glasgowi* * (1)
*Cosmolaelaps*	*C. retirugi* (13), *C. yeruiyuae* (9), *C. xiajiangensis* (1), *C. miles* (1), *C. hrdyiSam* (1)
*Androlaelaps*	*A. hsui* (52), *A. singularis* (6), *A. pavlovskii* (1)
*Hirstionyssus*	*H. musculi* (39)
*Hypoaspis*	*H. ovatus* (36)
*Haemogamasus*	*H. monticola* (2)
*Eulaelaps*	*E. substabularis* (1)
*Lasioseius*	*L. spatulus* (1)
**Macronyssidae**	66 individuals, 1 species, 1 genus
*Ornithonyssus*	*O. bacoti* * (66)
**Dermanyssidae**	13 individuals, 1 species, 1 genus
*Echinonyssus*	*E. nasatu* (13)

Annotation: The species marked with “*” are vector mite species, which can serve as vectors or potential vectors of hemorrhagic fever with renal syndrome (HFRS).

**Table 3:** Infestation indexes of dominant gamasid mite species at Waxi Village of Binchuan County, Yunnan Province of southwest China (2020–2021).

Gamasid Mite Species	No. of Hosts		No. and Constituent Ratios (*C_r_*) of Gamasid Mites		Infestation Indexes of Gamasid Mites		
	Hosts Examined	Hosts Infested	No.	*C_r_*, %	*P_M_*, %	*MA*	*MI*
*L. algericus*	1329	357	8262	68.42	26.86	6.22	23.14
*L. guizhouensis*	1329	36	1278	10.58	2.71	0.96	35.50
*L. nuttalli*	1329	155	1202	9.95	11.66	0.90	7.75

**Table 4:** Seasonal distribution of total trapped small mammal hosts and the 612 valid hosts infested with gamasid mites.

Season	Total Small Mammal Hosts Captured	Numbers of Valid Hosts and Their Gamasid Mites	
		No. of Valid Hosts	No. of Gamasid Mites
Spring	331	159	2985
Summer	331	179	4731
Autumn	326	150	3160
Winter	341	124	1199
Total	1329	612	12,075

Annotation: Total captured small mammals = 1329. The valid hosts (*N* = 612) represent only those individual hosts currently infested with gamasid mites, which provide meaningful data for further parasite community and network analyses (717 uninfested hosts were excluded from the community dataset).

**Table 5:** Statistics on *α*-diversity indices of gamasid mite community on small mammals in different months at Waxi Village of Binchuan County, Yunnan Province of southwest China (2020–2021).

	Month	Species Richness	Margalef	Gini–Simpson	Shannon–Wiener Index	Pielou’s Evenness
1	Jan	13	1.91	0.73	1.68	0.65
2	Feb	9	1.37	0.72	1.49	0.68
3	Mar	11	1.46	0.64	1.24	0.52
4	Apr	7	0.88	0.29	0.63	0.32
5	May	10	1.29	0.55	1.18	0.51
6	Jun	14	1.68	0.34	0.70	0.27
7	Jul	11	1.39	0.38	0.95	0.39
8	Aug	14	1.86	0.44	1.02	0.39
9	Sep	10	1.30	0.42	0.80	0.35
10	Oct	11	1.32	0.33	0.65	0.27
11	Nov	14	**2.41**	**0.79**	**1.96**	**0.74**
12	Dec	7	1.03	0.41	0.88	0.45

**Table 6:** Calculation of hosts’ preference for gamasid mites at Waxi Village of Binchuan County, Yunnan Province of southwest China (2020–2021).

Host Name	Degree	Normalized Degree	Species Strength	Nested Rank	Species Specificity Index
*A. chevrieri*	18	0.545	8.245	0.091	0.768
*A. peninsulae*	12	0.364	2.733	0.273	0.622
*C. erythraeus*	3	0.091	2.918	0.727	0.965
*D. pernyi*	1	0.030	0.033	1.000	1.000
*E. miletus*	2	0.061	1.667	0.909	0.919
*M. caroli*	12	0.364	2.574	0.182	0.940
*M. cookii*	5	0.152	1.175	0.545	0.908
*M. pahari*	4	0.121	0.051	0.636	0.822
*N. confucianus*	7	0.212	2.118	0.364	0.600
*R. andamanensis*	20	0.606	9.413	0.000	0.714
*R. norvegicus*	7	0.212	1.069	0.455	0.874
*T. belangeri*	3	0.091	1.004	0.818	0.858

**Table 7:** Host specificity of gamasid mite species at Waxi Village of Binchuan County, Yunnan Province of southwest China (2020–2021).

Name of Mite	Degree	Normalized Degree	Species Strength	Nested Rank	Species Specificity Index
*L. algericus*	6	0.500	3.135	0.063	0.647
*L. chini*	1	0.083	0.918	0.625	1.000
*L. hongaiensis*	1	0.083	0.011	0.750	1.000
*H. musculi*	4	0.333	0.037	0.281	0.650
*L. nuttalli*	6	0.500	1.629	0.094	0.788
*L. echidninus*	3	0.250	0.371	0.406	0.693
*L. turkestanicus*	4	0.333	0.669	0.188	0.682
*L. fukienensis*	2	0.167	0.081	0.500	0.789
*C. retirugi*	4	0.333	0.077	0.344	0.526
*L. guizhouensis*	7	0.583	1.555	0.000	0.678
*H. ovatus*	4	0.333	0.066	0.313	0.743
*L. paucisetosa*	5	0.417	0.116	0.156	0.804
*L. xingyiensis*	7	0.583	0.087	0.031	0.673
*L. taingueni*	5	0.417	0.205	0.125	0.680
*E. nasatus*	2	0.167	0.859	0.531	0.919
*O. bacoti*	3	0.250	0.032	0.438	0.761
*A. hsui*	4	0.333	0.026	0.250	0.516
*H. liae*	4	0.333	1.968	0.219	0.912
*C. yeruiyuae*	4	0.333	0.083	0.375	0.659
*H. monticola*	1	0.083	0.001	0.719	1.000
*C. miles*	1	0.083	0.001	0.781	1.000
*A. pavlovskii*	1	0.083	0.001	0.813	1.000
*E. substabularis*	1	0.083	0.000	0.844	1.000
*A. singularis*	3	0.250	0.003	0.469	0.674
*C. xiajiangensis*	1	0.083	0.017	0.875	1.000
*H. petauristae*	2	0.167	0.003	0.563	0.807
*H. glasgowi*	1	0.083	0.000	0.906	1.000
*H. chinensis*	1	0.083	0.001	0.688	1.000
*H. cordatus*	2	0.167	0.009	0.594	0.718
*L. hilaris*	1	0.083	0.017	0.656	1.000
*L. spatulus*	1	0.083	0.001	0.938	1.000
*C. hrdyi*	1	0.083	0.002	0.969	1.000
*H. orientalis*	1	0.083	0.017	1.000	1.000

**Table 8:** Capture numbers and percentage compositions of dominant small mammal hosts across four seasons in Waxi Village, Binchuan County, Yunnan Province, southwest China (2020–2021).

Host Species	Spring		Summer		Autumn		Winter		Total	
	NO.	*C_r_* (%)	NO.	*C_r_* (%)	NO.	*C_r_* (%)	NO.	*C_r_* (%)	NO.	*C_r_* (%)
*Rattus andamanensis*	64	4.82	67	5.04	135	10.16	100	7.52	366	27.54
*Apodemus chevrieri*	89	6.70	126	9.48	114	8.58	26	1.96	355	26.71
*Mus caroli*	84	6.32	105	7.90	17	1.28	87	6.55	293	22.05

## Data Availability

The experimental data used to support the findings of this study are available from the corresponding author upon request.
